# Colorectal Cancer Diagnosis: The Obstacles We Face in Determining a Non-Invasive Test and Current Advances in Biomarker Detection

**DOI:** 10.3390/cancers14081889

**Published:** 2022-04-08

**Authors:** Faddy Kamel, Khadiga Eltarhoni, Pasha Nisar, Mikhail Soloviev

**Affiliations:** 1 Department of Biological Sciences, Royal Holloway University of London, Egham, Surrey TW20 0EX, UK; faddy.kamel.2018@live.rhul.ac.uk (F.K.); khadiga.eltarhoni.2021@live.rhul.ac.uk (K.E.); 2 Ashford and St Peter’s Hospitals NHS Foundation Trust, Chertsey KT16 0RQ, UK

**Keywords:** colorectal cancer, biomarkers, cancer detection, cancer screening, cancer treatment

## Abstract

**Simple Summary:**

Colorectal cancer (CRC) is one of the most common cancers in the western world. CRC originates from precursor adenomatous polyps, which may over time develop into cancer. Endoscopic evaluation remains the gold-standard investigation for the disease. In the absence of molecular tools for early detection, the removal of neoplastic adenomas via polypectomy remains an important measure to prevent dysplastic adenomas from evolving into invasive carcinoma. Colonoscopy is an intrusive procedure that provides an uncomfortable experience for patients. Kits for testing for the presence of blood hemoglobin in the stool are now widely used, and DNA methylation-based detection kits have been approved in the USA for testing the stool and plasma, but few other molecular biomarkers have found their way into medical practice. This review summarizes current trends in the detection and screening of CRC and provides a definitive review of emerging molecular biomarkers for CRC.

**Abstract:**

Globally, colorectal cancer (CRC) is the third most common cancer, with 1.4 million new cases and over 700,000 deaths per annum. Despite being one of the most common cancers, few molecular approaches to detect CRC exist. Carcinoembryonic antigen (CEA) is a known serum biomarker that is used in CRC for monitoring disease recurrence or response to treatment. However, it can also be raised in multiple benign conditions, thus having no value in early detection or screening for CRC. Molecular biomarkers play an ever-increasing role in the diagnosis, prognosis, and outcome prediction of disease, however, only a limited number of biomarkers are available and none are suitable for early detection and screening of CRC. A PCR-based Epi proColon^®^ blood plasma test for the detection of methylated SEPT9 has been approved by the USFDA for CRC screening in the USA, alongside a stool test for methylated DNA from CRC cells. However, these are reserved for patients who decline traditional screening methods. There remains an urgent need for the development of non-invasive molecular biomarkers that are highly specific and sensitive to CRC and that can be used routinely for early detection and screening. A molecular approach to the discovery of CRC biomarkers focuses on the analysis of the transcriptome of cancer cells to identify differentially expressed genes and proteins. A systematic search of the literature yielded over 100 differentially expressed CRC molecular markers, of which the vast majority are overexpressed in CRC. In terms of function, they largely belong to biological pathways involved in cell division, regulation of gene expression, or cell proliferation, to name a few. This review evaluates the current methods used for CRC screening, current availability of biomarkers, and new advances within the field of biomarker detection for screening and early diagnosis of CRC.

## 1. Introduction

Colorectal cancer (CRC), also referred to as bowel cancer, is one of the most common cancers in the western world. Globally, it ranks as the third most common cancer, with 1.4 million new cases and causing over 700,000 deaths per annum [1]. Within the United Kingdom, for both males and females, it is also the third most commonly diagnosed cancer, with over 40,000 new cases every year [2,3,4,5,6,7,8,9,10]. There is a predicted continuing exponential rise in the total number of cases and an expectation of a 60% increase in the incidence by 2030, provoking a persistent drive to develop early diagnostic and screening techniques [11]. As many as 45% of patients diagnosed with CRC are estimated to die as a result of the disease worldwide [12]. CRC is more prevalent in countries of higher socioeconomic status, whereas developing regions show lower rates, with up to a ten-fold difference seen across regions [11]. The high prevalence of CRC in the western world is evident in the UK, where CRC is the second most common cause of cancer mortality.

CRC is heterogeneous in nature and it is widely accepted that most cases are sporadic (between 70 and 80%), whereas the remaining 20–30% are known to have a hereditary element [13]. Those rare cases that are hereditary, include cases due to either familial adenomatous polyposis (FAP), resulting from a defect in the adenomatous polyposis coli (APC) tumor suppressor gene and carrying a nearly 100% risk of colon cancer development [14], or the more common but less severe Lynch Syndrome, caused by mutations in DNA mismatch repair mechanisms (genes involved include MLHL, MSH2, MSH6, and PMS2) [15]. Both conditions increase the susceptibility of the affected individual to develop colorectal carcinoma [6]. Polyps are abnormal growths that protrude into the lumen of a hollow viscus. Within the colon and the rectum, they arise from the epithelium of the mucosa. There are various types of polyps described based on their macroscopic appearance of being sessile (flat) or pedunculated (stalked). Polyps can be further classified histologically into three main categories, of which neoplastic polyps (adenomas) are of the most importance to CRC. Whilst the vast majority of adenomas do not evolve into carcinoma, the great majority of CRC cases originate from adenomatous polyps [16]. The mechanisms that cause an adenoma to transform into a malignant tumor are broadly divided into three different types: (i) mutations of proto-oncogenes that cause transformation into oncogenes, (ii) mutations or deletions that reduce the activity of tumor suppressor genes, and (iii) mutations leading to the impairment of DNA mismatch repair [17]. The removal of neoplastic adenomas via polypectomy, before they evolve into cancerous carcinomas, is an important preventative measure to stop neoplastic adenomas from becoming malignant tumors [18].

## 2. Detection and Screening in Colorectal Cancer

The screening process for abnormal adenomas and early diagnosis of CRC is currently conducted through colonoscopies, which have been recognized as the ‘gold standard’ due to both their potential in the diagnosis and removal of adenomas [19]. Yet, colonoscopy is an uncomfortable and invasive investigation for patients, requiring bowel preparation from the day before and the patient on clear fluids only in the 12 h before the procedure. This is followed by bowel preparation, which is seldom a nice experience for any patient. Thus, many patients do not comply with the prospect of having to undergo such an investigation, which can be very uncomfortable, and in some instances, patients refuse to undergo the procedure [20]. Coupled with this, there is growing pressure on endoscopy units in the UK to deliver many urgent colonoscopies that are referred through the ‘Urgent Suspected Cancer’ (USC) pathway, meaning that the investigation must be carried out within two weeks of referral. Within the UK, the yield for CRC diagnosis from all patients that undergo a colonoscopy under the USC pathway is 3%, which is low [21]. There is also an associated financial burden on the National Health Service (NHS) of colonoscopies, with a cost of £460 per procedure, rising to £528 if biopsies are taken [22]. A combination of patient reluctance and low yield of diagnosis provokes a pressing need for a non-invasive or minimally-invasive biomarker test that can be made available to reduce the need for such an invasive investigation.

**Figure 1:**
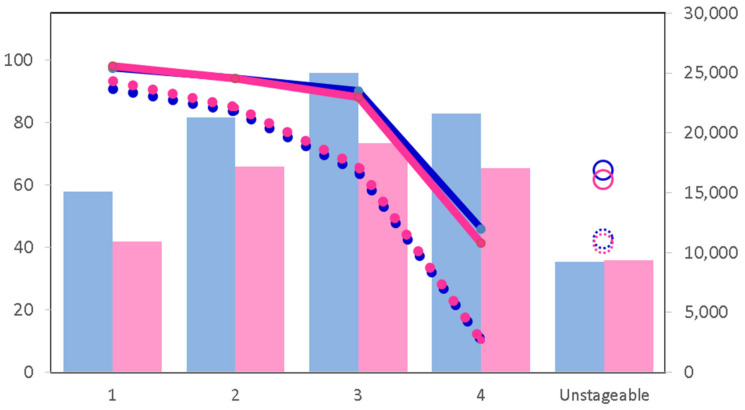
Bowel cancer survival by stage of disease at diagnosis. Solid blue and red lines denote one-year survival post-diagnosis for males and females, respectively. Open circles show survival rates (%) for males and females (blue and red, respectively) where no staging data are available. Dotted lines indicate five-year net survival. Numbers of cases are shown as blue bars (males) and pink bars (females)—right vertical axis. Horizontal axis—stage at diagnosis. Data are from [9] and refer to adults diagnosed in 2013–2017 and followed up to 2018.

Early detection of CRC is intimately linked to higher survival rates from the disease, ranging from as high as a 90% five-year survival rate in stage 1 disease, to as low as 10% five-year survival in stage 4 disease [23]. CRC survival rates are high for the early stages of diagnosis, but both one-year and five-year survival rates reduce sharply for late diagnosis (e.g., stage 4, Figure 1) [24]. To alleviate this, there have been many attempts to enable the early detection of CRC, which is largely asymptomatic, to achieve higher survival rates and reduce the disease burden [25]. Proposed guidelines to reduce the incidence of CRC recommend that the screening procedure should be carried out initially at the age of 50 and then every 10 years thereafter [26]. With early screening, pre-malignant lesions such as adenomas can be detected. These can be removed endoscopically rather than via surgery, which is much less invasive and with a significantly lower rate of mortality and morbidity, and a lower overall effect on the quality of life [27]. The presence of polyps does not lead to a reduced life expectancy, unlike malignancy.

The current screening program within the UK for CRC involves patients being invited to provide a stool sample, which is then tested for the presence of blood in the stool in the form of fecal immunohistochemistry testing (FIT), where patients are invited on a two-yearly basis from the age of 60 to 74 [28]. A positive test triggers a referral for a colonoscopy to confirm the suspected diagnosis. When the fecal hemoglobin (f-Hb) cutoff level of 150 µg/g blood is used, the sensitivity is around 70.8%, with a high specificity of 95.6%; however, the specificity drops to around 64% when the cut off level is reduced to 2 µg/g blood [29]. Samples within the UK are commonly analyzed using the OC-Sensor™ kit. The recommended cutoff value for this test for the symptomatic population is an f-Hb of 10 µg/g feces (equivalent to 50 ng/mL) [30]. Other immunochemical diagnostic tests recommended in the UK by NICE [31] for testing for the presence of blood in the stool include the HM-JACKarc system (the recommended f-Hb cutoff value is 10 ng/mL, [32]), RIDASCREEN hemoglobin or RIDASCREEN hemoglobin/haptoglobin enzyme immunoassay (ELISA) tests (the recommended f-Hb cut-off value is 2 µg/g for either of the two tests, [33]), and Faecal Occult Blood (FOB) Gold system [34]. The FOB test does not specify a cutoff, which should be adjusted depending on the individual laboratory’s setup and needs to be determined experimentally. This test has a much lower specificity rate compared to other immunochemical tests, with a false negative result for cancer found in up to 35% of patients [7]. In certain parts of the UK, depending on available resources, patients are invited to undergo a one-time-only flexible sigmoidoscopy at the age of 55 [28]. In Germany, for example, guidelines are similar to those found in the UK with two-yearly FIT test screening, but beginning from the age of 50 up to 74 [35]. In the USA, the Centre for Disease Control (CDC) guidelines recommend screening for CRC between the ages of 45 and 75 with an annual FIT test and colonoscopy every 10 years for asymptomatic patients and those with a negative FIT test [36]. A study that compared the effectiveness and cost-effectiveness of FIT and colonoscopy with a multi-target stool DNA (MT-sDNA) test found that the latter is less effective and more costly when used for detecting CRC and polyps [37]. The ‘gold standard’ investigation for CRC worldwide remains a colonoscopy.

The current use of molecular biomarkers in the detection of CRC is extremely limited. The only serum biomarker for CRC tested for in the UK is carcinoembryonic antigen (CEA) (UniProtKB ID Q13982) [38]. However, CEA is not tested routinely in worldwide practice to aid in the diagnosis of CRC as levels may be raised in various other inflammatory conditions such as diverticulitis and inflammatory bowel disease [39]. CEA may also be raised in other cancers such as pancreatic cancer [40]. CEA thus has poor sensitivity but excellent specificity. Thus, CEA is tested for in patients that are known to have CRC, to either monitor their response to treatment after chemotherapy or to screen for recurrence of disease after treatment has been completed [41]. One limitation of CEA-based diagnostics is that there is no direct correlation between the degree of CEA upregulation and the prognosis or spread of the disease. No other suitable diagnostic molecular biomarker is currently used to screen for CRC. One other biomarker, KRAS (UniProtKB ID P01116), is used to guide adjuvant CRC therapy. Cases of CRC where mutations in the KRAS gene are detected in the cancer cells are resistant to the anti-EFGR chemotherapy drug cetuximab, thus precluding the use of this treatment modality [42].

## 3. Pathogenesis of Colorectal Cancer

Three main distinct pathways lead to the carcinogenesis of CRC. They are chromosomal instability (CIN), microsatellite instability (MSI), and CpG methylator phenotype (CIMP). Within each pathway, there are different mechanisms and degrees of genetic instability, and tumors will often show evidence of multiple mechanisms [43].

**Figure 2:**
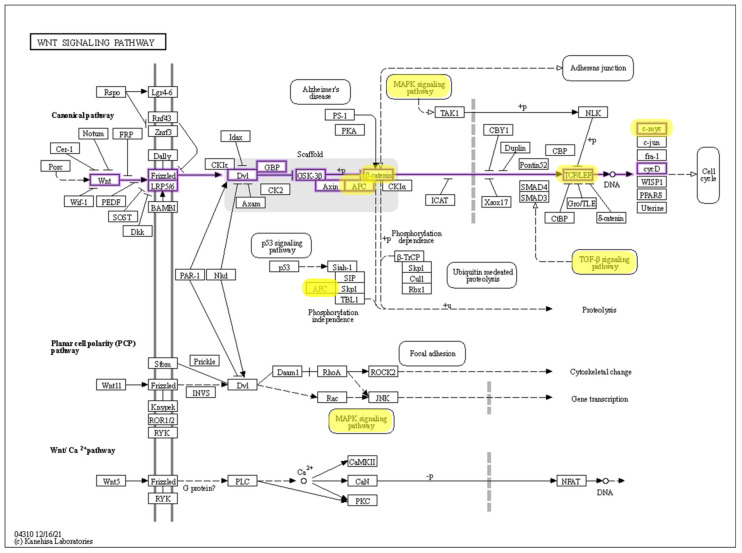
Wnt signaling pathway. Three pathways are shown—canonical, planar cell polarity (PCP), and Wnt/Ca^2+^. Molecules, pathways, and interactions implicated in CRC are highlighted. Reproduced from [44] with permission.

The wingless and Int-1 (Wnt) signaling pathway [44,45] is involved in tissue hemostasis and repair, and is thought to show levels of increased activation in almost all cases of CRC [46], as well as being implicated in a multitude of other cancers [47]. Under normal conditions, the Wnt signal transduction pathway uses transmembrane receptors to elicit intracellular signals for the transcription of target genes. In a normal colon, such signaling is important as colon epithelial cells are regularly damaged by the mechanisms of motility, and therefore, the continuous regeneration of crypt cells is essential. This process is tightly regulated by the adenomatous polyposis coli (APC) protein, which regulates the breakdown of β-catenin, an important transcription factor for the proliferation of crypt epithelial cells. [48]. However, in the chromosome instability pathway, the APC gene and its transcribed protein are dysfunctional. Therefore, an APC complex cannot form with the cytosolic β-catenin, which causes β-catenin to accumulate in the cytosol and eventually translocate into the nucleus, where β-catenin binds to a TCF transcription factor and transcribes the MYC protein, activating its associated MYC pathway and initiating proliferation. Following the initial mutations of APC, a coupled transformation occurs of the proto-oncogene KRAS, in the chromosome instability pathway, or BRAF, in the microsatellite pathway, into active oncogenes, whereby changes in intracellular signal transduction in the MAPK pathway lead to changes in successive pathways (PI3K and TGF-β), inducing excessive growth signals and proliferation [16] (Figure 2). It is important to note that the accumulation of many more genetic mutations is required for the progression of carcinogenesis, not purely APC, KRAS, and BRAF. However, the order in which these mutations occur does not determine the initiation of carcinogenesis; instead, it is the occurrence of 15 recognized ‘drivers’ that are associated with activating carcinogenesis pathways that form from adenomas. The idea that there is a linear defined sequence that leads to the development of colorectal carcinomas is rather limited [48].

**Figure 3:**
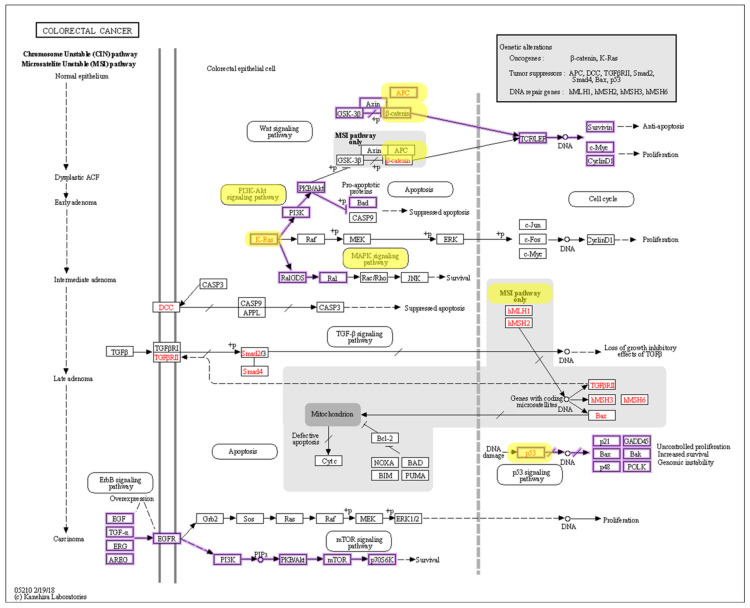
Colorectal cancer pathways. Molecules, pathways, and interactions implicated in CRC are highlighted. Reproduced from [49] with permission.

The chromosomal instability (CIN) [49] pathway is thought to be the classical pathway of carcinogenesis of CRC because it can be present in up to 85% of all cases of CRC [50]. This pathway is characterized by abnormal gains and losses of large parts of, or in some cases, whole chromosomes. That leads to aneuploidy, loss of heterozygosity, and mutations that cause loss of function of TP53, a tumor suppressor gene that encodes for P53 protein [43,51], which itself is the main checkpoint in the cell cycle. Two different pathways within CIN are thought to lead to this. These include the APC, KRAS, and PI3K pathways. APC mutations lead to the translocation of β-catenin to the nucleus of the cell, which then promotes transcription of the genes that are involved in carcinogenesis. Mutations within KRAS and PI3K, however, are known to cause constant activation of MAP kinase, which, in turn, leads to increased cell proliferation [52] (Figure 3).

The microsatellite instability (MSI) pathway [49] involves a loss of DNA mismatch repair (MMR) mechanisms. Tumors that lack MMR show a decreased ability to repair short strains of DNA or tandem repeats, resulting in mutations accumulating in these regions. The mutations that accumulate can affect coding regions of both oncogenes and tumor suppressor genes, as well as non-coding regions. Mutations leading to MSI can be sporadic or germinal as seen in Lynch syndrome [53]. The two most common mutated proteins involved in this pathway are MSH2 and MLH1, which account for up to 3% of all CRC [50] (Figure 3).

The CpG island methylator phenotype (CIMP) is present in almost all cases of CRC and is thought to work in conjunction with the other main pathways described above. Tumors that exhibit the CIMP phenotype, found mainly in serrated polyps [48,50,54], have an exceptionally high frequency of hypermethylated CpG dinucleotides, especially in the DNA of tumor suppressor genes, leading to the promotion of oncogenesis.

As genetic mutations continue to accumulate within these three main pathways of carcinogenesis of CRC, in which oncogene activity is initiated and tumor-suppressor gene activity is inhibited, the neoplastic adenoma continues to advance, leading to a dysplastic polyp. The extent of dysplasia and size of the polyp correlate with the malignant potential of the cell, whereby the greater the extent of dysplasia, the greater the malignant potential [55]. Eventually, the continuous intracellular signaling for the proliferation of epithelial cells leads to the penetration of the adenoma into the submucosa. At this point of penetration, the adenoma is thought to have transformed into a cancerous malignant tumor [56]. On achieving malignancy, the cellular structure and characteristics of these cells differ from their parent cells massively, with irregular nuclei and an ability to metastasize into other organs.

Other mechanisms in the pathogenesis of CRC that do not fit into the classical pathways have been described in the recent literature. The first of these concerns small non-coding RNAs (microRNAs) that are involved in many cellular functions, including regulation of tumor repressor genes and oncogene expression levels, the cell cycle, and cell apoptosis [57]. Studies have shown that microRNAs do initiate the pathogenesis of cancer by allowing over-growth of cells through the dysregulation of cell proliferation [58,59].

Other, albeit unproved pathways leading to CRC relate to adiponectin and IL-6. Adiponectin (GBP-28) is a protein hormone found in both white and brown adipose tissue and involved in the regulation of glucose levels as well as the breakdown of fatty acids [60]. It has three main polymeric forms: the low- (LMW), middle- (MMW), and high-molecular-weight complexes (HMW) [61]. However, the reported expression levels of adiponectin vary. One study of CRC tissue specimens reported higher expression of the AdipoR1 receptor and adiponectin by about 60% compared to normal mucosa. It is thought that this increased expression level could be a mechanism by which cancer cells achieve the increased oxidative metabolism that is needed to sustain increased cell proliferation [62]. Another study, however, has shown adiponectin to have lower expression levels in cancer tissue when compared to normal tissue [63].

Furthermore, adiponectin activates protein kinase A (PKA), which, in turn, leads to increased activation of AMP-activated protein kinase (AMPK) and decreased activation of AKT [64]. The AMPK pathway is involved in the regulation of the cell cycle, which is carried out by modulation of transcription factors, specifically including p53, which is a known tumor-suppressor gene [65]. Thus, any change in the expression levels of the AMPK pathway can lead to carcinogenesis. One function of adiponectin is to improve sensitization of the body to insulin, requiring an increased surge in insulin release to counter this. Insulin itself leads to decreased expression of the insulin-like growth factor binding protein-1 (IGFBP-1), which, in turn, causes a lowering in the circulating levels of insulin-like growth factor-1 (IGF-1). Lower levels of IGFBP-1 and IGF-1 were correlated with increased risks of CRC carcinogenesis [66]. The LMW complexes of adiponectin possess potent anti-inflammatory properties and thus underexpression will lead to increased inflammation and release of cytokines such as interleukin-6 (IL-6). Interleukin-6 (IL-6) has been shown to correlate with the pathogenesis of CRC, occurring in patients who are clinically obese (BMI > 30) [67]. The mechanism is thought to be related to the production of cytokines such as IL-6, which is elevated in a pro-inflammatory state that is induced by obesity. The mechanism of this stems from the hypoxia and death of adipocytes within adipose tissue, leading to immune cell infiltration and increased lipolysis. This, in turn, stimulates Toll-like receptor 4 (TLR4) on immune cells, leading to activation of pro-inflammatory signaling pathways and overexpression of cytokines including IL-6 [68]. The local inflammation in adipose tissue directly favors carcinogenesis systemically by causing underexpression of adiponectin. Increased levels of IL-6 have been shown to directly correlate with the TNM classification of CRC, i.e., higher levels of IL-6 are consistent with a higher TNM staging of CRC [69]. The risk of CRC carcinogenesis is increased in other inflammatory conditions linked to IL-6 such as Crohn’s disease and ulcerative colitis [70]. It is the prolonged inflammatory state that leads to the production of acute-phase proteins. As with adiponectin, this pro-inflammatory state leads to lower concentrations of IGFBP-1 and IGF-1, which as discussed previously, show increased risks of carcinogenesis of CRC.

## 4. Obstacles and Limitations to the Use of Biomarkers as a Screening Tool

Many serum biomarkers that are currently used for screening, diagnosis, or monitoring the progression of various cancers suffer the same issues as described with those of CEA, in that they have limited specificity and as some can be associated with various inflammatory processes within the body. For example, CEA can be raised in diverticulitis and inflammatory bowel disease [39]. CA19-9 (UniProtKB ID Q969X2), also known as carbohydrate antigen 19-9, is a tetra-saccharide that attaches to the O-glycans on the cell surface; it is commonly used as a tumor marker for pancreatic cancer [71]. CA19-9, however, can also be raised in other malignancies such as liver, gallbladder, and CRC [72]. CA19-9 may also be raised in benign inflammatory conditions of the biliary system such as hepatitis, cholecystitis, and obstructive jaundice [73]. CA125 (UniProtKB ID Q8WX17), also known as mucin 16, is a glycoprotein within the mucin family, and when levels are raised above 35 units/mL, there is an 80% chance of the presence of ovarian cancer in a patient [74]. However, it can also be raised in various benign inflammatory conditions such as endometriosis and pelvic inflammatory disease and so has no role in screening for ovarian cancer [75]. Thus, finding a biomarker that is specific to only one type of cancer has proven difficult over time. CA19-9 can be raised in multiple malignancies, and both CA19-9 and CA125 can be raised in benign inflammatory conditions. This reduces their usefulness as screening tools for cancer. This issue is shared by other tumor markers that are commonly associated with various cancers and remains the main obstacle that is yet to be overcome in the discovery of a serum biomarker that can be used as an accurate diagnostic screening tool for cancer. Another representative example of a less than perfect marker is prostate-specific antigen (PSA) (UniProtKB P07288), which is a glycoprotein enzyme used as a biomarker for screening prostate cancer. Higher levels above 3 ng/mL indicate up to a 60% likelihood of having prostate cancer, whereas a normal result of less than 3 ng/mL confers an 85% probability of not having prostate cancer [76]. PSA tests return many false-positive results and it is not a perfect screening tool for prostate cancer. Trauma to the prostate with the digital examination or urinary catheterization could also lead to a transient rise in PSA, fueling false-positive results [77].

In the USA, the American Cancer Society (ACS) and the Centers for Disease Control and Prevention guidelines recommend screening for CRC via either a FIT stool test, a multi-targeted DNA stool test, or colonoscopy [36,78]. In Germany, France, and Denmark, screening is also carried out using the FIT stool test and colonoscopy [79,80,81]. In the United Kingdom, no biomarkers have been approved for use as a screening tool to detect early CRC, which in many cases, will be asymptomatic. The National Institute of Clinical Excellence (NICE) and the Association of Coloproctology (ACPGBI) advocate for the use of the FIT test to screen patients who would need urgent endoscopic or radiological intervention [82,83]. Yet, no guidelines or approval have been given to any use of biomarkers in screening and early detection for CRC, and the use of CEA is limited to monitoring of treatment and observation of recurrent disease.

## 5. Current Advances in CRC Biomarker Detection

### 5.1. DNA-Based Molecular Markers and Tests

**Figure 4:**
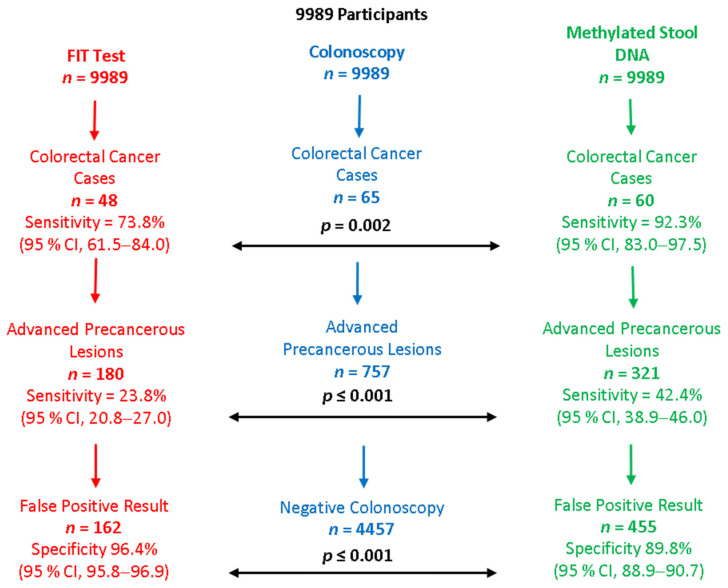
Summary of the main results of a study comparing FIT stool test to methylated DNA stool test for the detection of colorectal cancer [84].

Several recent studies have looked at testing DNA in feces, looking specifically for biomarkers in cells originating from colonic neoplasms. These studies have concentrated in some instances specifically on methylated DNA in the stool. One such study used a combined stool FIT and multi-targeted stool DNA test (mt-DNA). The mt-DNA tests relied on quantitative real-time PCR of bisulfite-converted DNA for the detection of hypermethylated NDRG4 and BMP3 gene promoters, for KRAS gene mutations, and using β-actin as an internal DNA reference. Regression analysis was then used to combine these data with the results of the stool hemoglobin component to yield a composite score, which was compared to a traditional stool FIT test that has been established for use in CRC screening [84]. The study involved nearly 10,000 patients, for whom the average risk of CRC was estimated. The stool DNA test was significantly better at detecting CRC than FIT (92.3% vs. 73.8%, *p* = 0.002) and advanced precancerous lesions including advanced polyps (42.4% vs. 23.8%, *p* < 0.001). However, there were more false-positive results than with FIT [84] (Figure 4 summarizes the study findings). The use of the mt-DNA test was approved for clinical use by the USFDA in 2014. A more recent retrospective cohort study [85] confirmed the ability of the mt-SDBA test to detect early-stage cancers (18% tested positive, with fewer than 1% having colorectal cancer and 60% having adenomas), though there were high false-positive rates (39% deemed false-positive) [85]. Other studies have also looked at testing stool for DNA, showing good potential for use in screening [86,87]. The multi-targeted stool DNA test has been adopted for use and forms part of the clinical guidelines for screening for CRC in the USA [36,78,88].

Recently, another serum blood test was developed in the USA, named the Epi proColon^®^. This is a serum blood test that with the aid of real-time PCR assays, detects the presence of methylated SEPT9 (mSEPT9), which is a known biomarker for CRC [89]. The overall sensitivity of the test is relatively low at detecting CRC, at 68.2% across all stages, with a specificity of only 79.1% [90]. That makes Epi proColon^®^ inferior to both colonoscopy and FIT. Furthermore, the Epi proColon^®^ test has a relatively high false-positive rate of around 12% and an overall poor sensitivity for precancerous adenoma lesions [91]. The test has been approved by the FDA for use only in patients who refuse to partake in traditional screening and is not part of any clinical guidelines for the screening of CRC in the USA [92]. The detection of mSEPT9 DNA can also be involved in other malignancies, including those of the urinary tract [93], brain [94], ovaries [95], breasts [96], and for leukemia [97]. Being minimally invasive, generally acceptable, and easy for patients, the mSEPT9-based serum test (Epi proColon^®^) has some advantages as a screening tool for CRC. From the patients’ perspective, Epi proColon^®^ provides a more appealing option and seems to be no different from other blood tests taken for any other reason, meaning some patients prefer this alternative to handling their stool samples for a FIT test. Such patients’ hesitance invariably leads to a lower engagement rate. The use of Epi proColon^®^ as an alternative testing procedure is better than not using any test, and therefore, the use of this test increases CRC screening rates and population coverage. However, given the relatively low specificity rate for ruling out CRC, and the lower sensitivity of the mSEPT9 test for early CRC stages, this test could not be used as a sole tool for CRC screening, but would need to be in conjunction with a detailed patient history and examination. Furthermore, patients with a negative test who still manifest symptoms akin to CRC, as well as patients with a positive mSEPT9 test, will still require endoscopic examination of the colon. Therefore, the existing versions of the Epi proColon^®^ mSEPT9 test cannot replace other existing tools as a sole screening tool for CRC detection. However, combining mSEPT9 with FIT or FOB does improve the diagnostic sensitivity, and in combination with colonoscopy, reduces CRC mortality.

Syndecan-2 (SDC2, UniProtKB ID P34741) is a transmembrane protein that is known to be involved in many cellular processes associated with carcinogenesis including cell proliferation, angiogenesis, and cell migration [98]. Aberrant methylation of the SDC2 gene has also been shown to be involved in the pathogenesis of CRC. Its detection is possible from a tissue, blood, or stool sample, and the marker has been shown largely in late-stage III/IV disease [99]. A serum blood test has been developed, which looks at the methylation of a combination of Sept9 and SDC2 for use in the early detection of CRC, which is still awaiting approval. Studies have shown promising results, with an overall sensitivity of up to 80% and specificity of 92% [100]. Further improvements to the sensitivity of CRC detection with SDC2 methylation assays could be achieved by combined detection of hypermethylated TFPI2 and hypomethylated SDC2 [99,101]. Other DNA methylation markers linked to CRC include SFRP2 (obtainable from stool samples, with a sensitivity and specificity of 77%, [102]), VIM (obtainable from the serum, with a sensitivity of detection of 36.1%, 45.2%, 55.4%, and 85.7% for CRC stages 1 to 4, respectively, when used in combination with traditional CEA analysis, [103,104]), FBN2, and TCERG1 (sensitivities of 86% and 99%, respectively, if detected from tumor tissue) [105]. Over the last decade, many research publications have reported other promising methylated DNAs detectable in the blood as diagnostic, prognostic, and predictive markers of CRC (reviewed in [106]). Whilst DNA methylation represents a phenomenon common to many cancers, is detectable using a modified PCR-based approach, and provides a more stable type of molecular marker compared to, e.g., circulating RNA, all such normally intracellular molecules require mechanical rupture of malignant cells following their necrosis or apoptosis. Therefore, the mSEPT9 test and any other similar future tests aiming to detect methylated ctDNA will inevitably have limited sensitivity to early-stage CRC and pre-cancerous states such as advanced adenomas.

### 5.2. Circulating Tumor Cells

Circulating tumor cells (CTCs) provide another promising avenue explored with the view of early detection of CRC. However, one limitation of using CTCs is their low abundance (of the order of 10^9^ fewer than red blood cells) and the consequent need for their enrichment and capture, as well as their physical and biochemical heterogeneity [107]. Whilst a wide range of methods relying on the physical properties of CTCs have been reported (density, size, deformability, electrophoretic properties), affinity-based capture, such as positive selection for EpCAM or negative selection for CD45, remain the preferred methods of CTC capture and enrichment [108]. Among the advantages of relying on CTC analysis is the ability to generate insights into the complete transcriptome of individual CTCs, to better understand their unique molecular phenotypes and accurately identify their molecular pathological subtype [109], chemoresistance, or metastatic progression [110]. The avenue that has been explored thus far is linked to CTCs arising from KRAS gene mutations within CRC cells. These mutations occur in around 45% of all cases of CRC, and their detection can be achieved using, for example, digital droplet PCR (ddPCR) techniques on serum blood samples [111]. KRAS mutations are important in CRC as it is one of the downstream effectors of the EGFR pathway, which is known to be involved in the pathogenesis of CRC [112]. CTC detection provides a sensitivity of around 83% for the mutations found in the serum compared to those found in the actual tumor, meaning there is potential for future opportunities for early detection and monitoring of patients with CRC [111].

### 5.3. microRNAs and Other Non-Coding RNAs

Along with DNA methylation, microRNAs (miRNA) represent one of the key existing epigenetic mechanisms responsible for the regulation of gene expression, and therefore, gene function. There has been vast interest in the potential use of miRNA markers in the early screening and diagnosis of CRC, although these remain in the early stages of trials. miRNAs are typically detected and quantified via reverse transcription-quantitative PCR (RT-qPCR), RNA sequencing (RNA-Seq), or using microarrays. Recent studies have shown that the overall sensitivities can be around 76%, with a similar specificity level, which means there is potential for future use in screening and early detection. There have been numerous candidate miRNAs including miR-21 [113] and miR-23a [114]. Another miRNA candidate, miR-378 has been found to affect signaling pathways that control processes such as cell proliferation and apoptosis, specifically in stage II CRC [115]. Achieving detection of stage II cancer is an impressive accomplishment, but the test is likely to miss patients with stage I CRC at the time of testing, which would rule out the advantage of early detection of CRC via screening. Other miRNAs of interest are miR-135a and miR-135ve, which affect APC gene expression and Wnt pathway activity, both of which play a role in the pathogenesis of CRC [116]. In another study, a panel of six miRNAs was developed for studying CRC recurrence. Three miRNAs were significantly decreased (miR-93, miR-195, and let-7b) and three were significantly increased (miR-7, miR-141, and miR-494) in patients with early relapse and were also associated with decreased survival rates [117]. Another recent study looked at serum miR-92a-1, which showed a sensitivity of 81.8% and specificity of 95.6% [118]. This represents great potential; however, this was a relatively small study on 148 patients, and thus, there is not enough evidence yet for it to be used clinically on a wide scale. A different miR has also been described, namely miR-30a-35p, which was shown to be downregulated in patients with CRC with a relatively high value of area on a receiver operating characteristic (ROC) curve, giving it high potential to be used as a screening tool in the future. However, sensitivity and specificity tests have yet to be carried out [119].

Another group of circulating markers includes other non-coding RNAs, such as long non-coding RNAs (lncRNAs). As an example, lncRNA differentiation antagonizing non-protein coding RNA (DANCR) was upregulated in CRC serum samples, and its level correlated with the clinicopathological features of the CRC patients [120]. Another example of circulating lncRNA is serum NEAT1, which was identified as an independent prognostic factor for CRC and also as a marker to help differentiate metastatic CRC from non-metastatic CRC [121]. Other known examples of lncRNAs in CRC were characterized from cancer cells and tissues, e.g., CCAT1, CCAT12, CASC11, CRNDE, GAS5, H19, HOTAIR, PCAT 1, RAMS11, and UCA1. Circular RNAs (circRNAs) represent another relatively stable molecular species detectable in serum. Due to their covalently-closed loop structure, these single-stranded non-coding RNAs are particularly stable and provide potentially useful markers, such as, for example, circ-PNN (hsa_circ_0101802) [122].

### 5.4. Differential Gene and Protein Expression in CRC

Another marker research area that has attracted much attention concerns the analysis of transcriptome alterations in CRC to identify differentially expressed genes and proteins. Identification of differentially expressed genes has the potential to reveal molecular markets, both at the mRNA and protein levels, involved in tumor development and progression, as well as markers suitable for cancer detection. Increasing numbers of promising CRC molecular markers and targets are being discovered and reported in the literature, and these can be generally divided into four major categories: (1) markers associated with a poor or favorable prognosis; (2) markers associated with a high relapse rate in CRC; (3) markers for CRC resistance to treatment modalities, and (4) potential targets for treatment. A systematic search of the recent literature yielded over 100 differentially expressed CRC molecular markers and targets, the vast majority of which are overexpressed in CRC, though a smaller number of markers are downregulated. In terms of function, these ~100 genes represent over 1000 various biological pathways, but some are strongly overrepresented in this selection. These include the cell division pathway, pathways representing regulation of gene expression, regulation of cell proliferation, positive regulation of transcription, G-protein coupled receptor signaling, the inflammatory response, signal transduction, and chemokine-mediated signaling, as well as negative regulation of apoptosis. All of the ~80 upregulated genes markers listed in Supplementary Tables S1 and S2 are potentially suitable for molecular detection of CRC, and in the majority of these genes, association with a poor prognosis has been reported. In addition, many of the overexpressed proteins in CRC have been suggested as potential treatment targets. For the 14 genes and their product proteins reported to be downregulated in CRC, their lower expression levels correlate with a poor prognosis, while a lesser degree of downregulation is linked to a better prognosis (Tables S2 and S3).

Metastasis and the relapse rate are vital in cancer diagnosis, and any biomarker that could provide an index for these factors would prove highly beneficial. Of particular interest might be stromal cell-derived factor 1 (CXCL12), cyclin-dependent kinases regulatory subunit 2 (CKS2), metalloproteinase inhibitor 1 (TIMP1), centrosomal protein of 55 (CEP55), and guanine nucleotide-binding protein G(I)/G(S)/G(O) subunit gamma-2 (GNG2), for which overexpression has been linked to higher relapse rates [123,124,125,126,127,128,129,130]. There are several differentially expressed genes in CRC that are believed to be associated with the epigenetics of DNA and miRNA. For example, the upregulation of SLC10A1, MAPT, SHANK2, PTH1R, and C2, and the downregulation of CAB39, CFLAR, CTSC, THBS1, and TRAPPC3 have been proposed as markers of CRC metastasis in the liver [131].

Molecular biomarkers may also support quantitative analysis of CRC resistance to treatment modalities. Chemoresistance and radio-resistance reduce the effectiveness of treatment regimens and are difficult to anticipate in patients. Therefore, introducing a biomarker that indicates certain cancer sensitivities and guides the response to treatment is imperative. As an example, protein tyrosine kinase 6 (PTK6) is a protein kinase that in normal cells, functions as a cytoplasmic signal transducer. However, in CRC, the interaction between PTK6 and Janus kinase 2 (Jak2) promotes chemoresistance, and it has been proposed that adding a PTK6 inhibitor to the chemotherapy regimen may improve the chemosensitivity of CRC [132]. Enoyl-CoA hydratase 1 (ECHS1) is an enzyme that promotes the glycosylation of ceramide, which is believed to be a key step in chemotherapy resistance. Monitoring this marker may assist in the selection of appropriate patients for chemotherapy [133]. Another marker, N-MYC downstream-regulated gene 1 (NDRG1), a key regulator of a variety of cell growth regulatory processes and signaling pathways, was shown to enhance chemosensitivity by modulating EGFR trafficking in metastatic CRC [134]. DNA topoisomerase 2-alpha (TOP2A) is a nuclear decatenating enzyme that alters the DNA topology. Alterations to TOP2A expression and mutations are associated with more advanced CRC and alteration of the cancer response to chemoresistance [135].

Overexpressed CRC proteins may provide convenient targets for CRC treatment. As an example, epiregulin (EREG) is a peptide hormone, a member of the epidermal growth factor (EGF) family. EREG is associated with the demethylation of two promoter locations, which, in turn, leads to upregulation of the EGF receptor’s phosphorylation, resulting in the development of adenocarcinoma. Upregulation of EGF crypt-cell-to-CRC-transformation is one of the steps that occur during the adenoma-carcinoma transition stage [136]. Yes-associated protein 1 (YAP1) has been found to have the same effect and is one of the main effectors of the Hippo pathway. It is known to have an association with several cancers, including CRCs, and the levels of YAP1 in the cytoplasm of CRC cells are believed to be linked to patient survival. The higher the levels, the poorer the prognosis [137]. Another recently reported serum marker is angiogenin. It has a sensitivity of 66.2% and specificity of 64.9% at ruling out CRC [138]. However, the specificity rate remains too low for it to be used as a screening tool for the early detection of CRC.

## 6. Conclusions

Current advances in biomarker use for early detection of CRC have shown great potential in past years, and even though use of the Epi proColon^®^ blood test has been approved for use by the FDA, it is still only reserved for patients who refuse to participate in traditional screening for CRC. Furthermore, it is not included in any clinical guidelines, which are what the vast majority of clinicians will follow, as evidence-based practice is the gold-standard of treatment for patients. Early detection of cancer facilitates treatment and improves survival. Molecular biomarkers play an ever-increasing role in modern diagnosis, prognosis, and outcome prediction of disease; however, only a limited number of validated marker molecules are available and none are suitable for early non-invasive detection and molecular diagnosis of cancer. Methylation of DNA remains the most promising molecular mechanism for the discovery of biomarkers for CRC screening and early detection.

Currently, the only biomarker used in the detection and screening of early CRC is that of the multi-targeted stool DNA testing in the USA. However, this relatively new tool has not been widely adopted yet as it is inferior to the use of FIT testing, which has been widely adopted across the USA and Europe. CEA remains the only known serum blood biomarker that is routinely used in CRC; however, it is unsuitable for early detection and screening. The advent of mSEPT9 DNA serum testing has provided a potential new screening tool for CRC, but it remains a third-line alternative for patients who do not want to participate in traditional screening and given that more accuracy is still needed.

Along with multi-targeted stool DNA, mSEPT9 DNA serum testing is in use only in the USA. There still remains a large unmet need for non-invasive biomarkers to be used as accurate screening tools for the early detection of CRC. The reasons for this include but are not limited to cost, resources, and also patient preference as many patients do not wish to undergo the grueling process of bowel preparation before a colonoscopy, which in itself is very invasive. There still exists a need for a non-invasive screening tool with the highest possible rate of determining true negatives, to allow clinicians to conclude with high confidence and solely based on such a test that the patient does not have CRC. Such a screening tool does not yet exist. The existing molecular markers utilized in stool and serum tests, and to a lesser degree the immunoaffinity-based FIT test, are suitable for identifying low CRC risk, but do not provide the high level of specificity needed for an accurate screening tool. Patients must still be assessed for symptoms as the tests alone cannot rule out the presence of CRC, and symptomatic patients still require an endoscopic investigation even with a negative test. If the ideal test did exist with very high specificity, then a negative result would enable clinicians to discharge a patient from an urgent suspected cancer pathway and reassure them. Yet, such tests do not currently exist.

The emerging field of cancer epigenomics is producing an increasing number of biomarkers such as DNA methylation and miRNA, not only for cancer detection, treatment selection, and disease monitoring but also for potential cancer therapy through epigenetic regulation of gene function. Another distinct multidisciplinary area of research combines multiple ‘omics’ approaches with data analysis sciences to make sense of the increasingly enormous knowledge of gene and protein expression. Combined with the recent worldwide re-emergence of qPCR-based nucleic acid detection and immunoaffinity protein assays, the availability of the growing number of molecular biomarkers provides a unique opportunity to modernize the current arsenal of CRC detection and testing.

New methods are needed to predict and test potential biomarkers for use in screening and early detection of CRC. Whilst mSEPT9 and other methylated DNA have potential, they still do not provide the accuracy needed for a clinician to convincingly rule out the presence of CRC based on a negative test. The main criteria for a better test for screening CRC can be split into two different categories—the patient’s perspective and the clinician’s perspective.

From the patient’s perspective, a serum blood test would be more acceptable than a stool test. Patients overwhelmingly prefer to not have to undergo a colonoscopy, which is very invasive and uncomfortable. The current FIT test, e.g., as used in the UK, requires the patient to handle their stool samples before returning them to the health provider. The procedure can be distressing for some patients and puts them off collecting and sending their samples, resulting in a low ~50% take-up in the population for screening currently. Therefore, a serum blood test, whether the existing mSEPT9 or one of other methylated DNA tests, which will undoubtedly be developed and introduced, is likely to be far more acceptable for patients and lead to higher uptake in the screening population. Ultimately, patients want and expect a simple point-of-care test that preferably does not require them to take their stool sample and that can definitively rule out CRC if negative. A quick turnaround of results is also expected, especially in cases of suspected cancer.

From the clinician’s perspective, the most important criterion to be met is that the specificity of the test is high, above 90% if possible. This will enable clinicians to confidently rule out CRC if the test is negative. Another current need relates to directing patients toward a specific therapy or evaluating its therapeutic efficiency. However, those tests would have to be different from CRC screening tools and would be performed on cancer tissue taken either from biopsies or after surgical resection, meaning different molecular marker-extraction protocols. It is likely that different markers, more suitable for cancer grading and stratification, would have to be investigated. The cost-effectiveness of such tests is another important consideration. All such tests would need to be developed in tandem with the discovery of new chemotherapy drugs that can prolong life and reduce the risk of recurrent disease.

PCR-based detection, amplification, or sequencing has in many cases already achieved its theoretical limit of single-molecule sensitivity; therefore, further improvements in the sensitivity and specificity of cancer screening and detection would have to come from introducing new or additional molecular markers, increasing the multi-targeting ranges of DNA- or RNA-based assays, expanding the range of epigenetic markers (DNA methylation, miRNAs, long and short non-coding RNAs, etc.), or conducting multi-organ testing. Ultimately, the development of screening tools together with the developments of AI to help assess and monitor the lifestyle and health will provide even bigger health benefits than limiting screening for disease diagnosis only. Further automation and AI developments should help develop CT-based non-invasive screening alternatives.

In the short term, the largest improvements in molecular diagnostics are likely to come from even minor increases in the number of disease-specific markers and better ways of separating them from copious ‘biological noise’ present in typical samples, whether stool, blood, or any other physiological samples. Combining genetic markers with the analysis of patients’ proteomes for secreted proteins indicative of the early stages of malignant developments may provide immediate benefits since both genetic and immunoaffinity testing platforms are already widely available. Signaling and other secreted proteins may become detectable in physiological liquids (e.g., serum, urine) well before the appearance of genetic markers, which often require cell lysis for their release. Unfortunately, no technology is yet capable of detecting proteins at the same low level as achieved with PCR amplification-based DNA detection.

In terms of sensitivity, DNA-based tests, which rely on PCR amplification, generally provide better sensitivity in detecting molecular markers over the existing FIT or any other immunoaffinity-based tests, in general, though they come at higher costs. Yet, even the increased testing costs might become a preferred option for health providers if such costs are offset by the huge life- and cost-saving benefits of the early diagnosis of CRC. For now, fecal immunochemical and methylated serum DNA tests remain the only approved tests in wider medical practice, with fecal DNA testing coming of age as another screening tool. All existing molecular screening methods still need to be made more accurate by improving their sensitivity and specificity rates.

One other positive development is the recent explosive growth of PCR-based health-screening technologies (due to the recent growth of COVID-19 testing) and the continuing drop in the costs of large-scale DNA sequencing. These, together with a better general acceptance of molecular diagnostic technologies by the population, suggest promising new avenues for technological development in CRC screening. Therefore, combining PCR sensitivity with the ability to simultaneously detect multiple molecular markers derived from blood samples, rather than stool samples, whilst driving the overall cost of the analysis down, appears to be the preferred and most likely direction of travel in CRC and cancer screening, in general.

## Supplementary Materials

The following supporting information can be downloaded at: https://www.mdpi.com/article/10.3390/cancers14081889/s1, Table S1: Upregulated genes in CRC; Table S2: Top biological pathways among the 102 CRC markers; Table S3: Downregulated genes in CRC.
