# Supplementary material for: Colorectal Cancer Diagnosis: The Obstacles We Face in Determining a Non-Invasive Test and Current Advances in Biomarker Detection
Source: Cancers (Basel). 2022 Apr 8;14(8):1889. doi: 10.3390/cancers14081889 (PMC9029324; doi:10.3390/cancers14081889)
Supplement: Supplementary file 1 [file cancers-14-01889-s001.zip › cancers-1643277-supplementary.pdf]

Supplementary materials

Colorectal cancer diagnosis: the obstacles we face in determining a non-invasive test and current advances in biomarker detection

Faddy Kamel [1,2], Khadiga Eltarhoni [1,2], Pasha Nisar [2,*] and Mikhail Soloviev [1,*]

[1] Department of Biological Sciences, Royal Holloway University of London, Egham, Surrey TW20 0EX, UK; faddy.kamel.2018@live.rhul.ac.uk (F.K.); khadiga.eltarhoni.2021@live.rhul.ac.uk (K.E.)
[2] Ashford and St Peter's Hospitals NHS Foundation Trust, Chertsey KT16 0RQ, UK
* Correspondence: pasha.nisar@nhs.net (P.N.); mikhail.soloviev@rhul.ac.uk (M.S.)

Table S1. Upregulated genes in CRC.

Gene	Protein	UniProt ID	Protein Name
ECHS1	Enoyl-CoA hydratase, mitochondrial	P30084	(ECHM_HUMAN)
TOP2A	DNA topoisomerase 2-alpha	P11388	(TOP2A_HUMAN)
CXCL12	Stromal cell-derived factor 1	P48061	(SDF1_HUMAN)
CKS2	Cyclin-dependent kinases regulatory subunit 2	P33552	(CKS2_HUMAN)
TTK	Dual specificity protein kinase TTK	P33981	(TTK_HUMAN)
NHP2	H/ACA ribonucleoprotein complex subunit 2	Q9NX24	(NHP2_HUMAN)
RAN	GTP-binding nuclear protein Ran	P62826	(RAN_HUMAN)
FOXO1	Forkhead box protein O1	Q12778	(FOXO1_HUMAN)
CDC6	Cell division control protein 6 homolog	Q99741	(CDC6_HUMAN)
YAP1	Transcriptional coactivator YAP1	P46937	(YAP1_HUMAN)
CDK1	Cyclin-dependent kinase 1	P06493	(CDK1_HUMAN)
VEGFA	Vascular endothelial growth factor A	P15692	(VEGFA_HUMAN)
CCNB1	G2/mitotic-specific cyclin-B1	P14635	(CCNB1_HUMAN)
DLGAP5	Disks large-associated protein 5	Q15398	(DLGP5_HUMAN)
CXCL1	Growth-regulated alpha protein	P09341	(GROA_HUMAN)
ADCY9	Adenylate cyclase type 9	O60503	(ADCY9_HUMAN)
NEK2	Serine/threonine-protein kinase Nek2	P51955	(NEK2_HUMAN)
NOB1	RNA-binding protein NOB1	Q9ULX3	(NOB1_HUMAN)
CCL21	C-C motif chemokine 21	O00585	(CCL21_HUMAN)
PTTG1	Securin	O95997	(PTTG1_HUMAN)
SERPINE1	Plasminogen activator inhibitor 1	P05121	(PAI1_HUMAN)
PLK1	Serine/threonine-protein kinase PLK1	P53350	(PLK1_HUMAN)
MYC	Myc proto-oncogene protein	P01106	(MYC_HUMAN)
RRM2	Ribonucleoside-diphosphate reductase subunit M2	P31350	(RIR2_HUMAN)
IGF1	Insulin-like growth factor I	P05019	(IGF1_HUMAN)
CHEK1	Serine/threonine-protein kinase Chk1	O14757	(CHK1_HUMAN)
GINS1	DNA replication complex GINS protein PSF1	Q14691	(PSF1_HUMAN)
NLRP7	NACHT, LRR and PYD domains-containing protein 7	Q8WX94	(NALP7_HUMAN)
EREG	Proepiregulin	O14944	(EREG_HUMAN)
UTP14A	U3 small nucleolar RNA-associated protein 14 homolog A	Q9BVJ6	(UT14A_HUMAN)
ANTXR1	Anthrax toxin receptor 1	Q9H6X2	(ANTR1_HUMAN)
ASF1A	Histone chaperone ASF1A	Q9Y294	(ASF1A_HUMAN)
TIMP1	Metalloproteinase inhibitor 1	P01033	(TIMP1_HUMAN)
CEP55	Centrosomal protein of 55 kDa	Q53EZ4	(CEP55_HUMAN)
SFTA2	Surfactant-associated protein 2	Q6UW10	(SFTA2_HUMAN)
AMH	Muellerian-inhibiting factor	P03971	(MIS_HUMAN)
MYH2	Myosin-2	Q9UKX2	(MYH2_HUMAN)

POU4F1	POU domain, class 4, transcription factor 1	Q01851	(PO4F1_HUMAN)
SIX4	Homeobox protein SIX4	Q9UIU6	(SIX4_HUMAN)
INHBA	Inhibin beta A chain	P08476	(INHBA_HUMAN)
ESM1	Endothelial cell-specific molecule 1	Q9NQ30	(ESM1_HUMAN)
SLC10A1	Sodium/bile acid cotransporter	Q14973	(NTCP_HUMAN)
MAPT	Microtubule-associated protein tau	P10636	(TAU_HUMAN)
DDIT4	DNA damage-inducible transcript 4 protein	Q9NX09	(DDIT4_HUMAN)
TPTE	Putative tyrosine-protein phosphatase TPTE	P56180	(TPTE_HUMAN)
PAICS	Multifunctional protein ADE2	P22234	(PUR6_HUMAN)
CCNA2	Cyclin-A2	P20248	(CCNA2_HUMAN)
PAX5	Paired box protein Pax-5	Q02548	(PAX5_HUMAN)
CXCL3	C-X-C motif chemokine 3	P19876	(CXCL3_HUMAN)
CXCL11	C-X-C motif chemokine 11	O14625	(CXL11_HUMAN)
PPBP	Platelet basic protein	P02775	(CXCL7_HUMAN)
CDC20	Cell division cycle protein 20 homolog	Q12834	(CDC20_HUMAN)
MCM4	DNA replication licensing factor MCM4	P33991	(MCM4_HUMAN)
CLDN1	Claudin-1	O95832	(CLD1_HUMAN)
SHANK2	SH3 and multiple ankyrin repeat domains protein 2	Q9UPX8	(SHAN2_HUMAN)
PTH1R	Parathyroid hormone/parathyroid hormone-related peptide receptor	Q03431	(PTH1R_HUMAN)
C2	Complement C2	P06681	(CO2_HUMAN)
TEAD4	Transcriptional enhancer factor TEF-3	Q15561	(TEAD4_HUMAN)
CXCL8	Interleukin-8	P10145	(IL8_HUMAN)
AQP8	Aquaporin-8	O94778	(AQP8_HUMAN)
PBX4	Pre-B-cell leukemia transcription factor 4	Q9BYU1	(PBX4_HUMAN)
POLR1B	DNA-directed RNA polymerase I subunit RPA2	Q9H9Y6	(RPA2_HUMAN)
BUB1	Mitotic checkpoint serine/threonine-protein kinase BUB1	O43683	(BUB1_HUMAN)
AURKA	Aurora kinase A	O14965	(AURKA_HUMAN)
GNG7	Guanine nucleotide-binding protein G(I)/G(S)/G(O) subunit gamma-7	O60262	(GBG7_HUMAN)
MAD2L1	Mitotic spindle assembly checkpoint protein MAD2A	Q13257	(MD2L1_HUMAN)
PTGS2	Prostaglandin G/H synthase 2	P35354	(PGH2_HUMAN)
NMU	Neuromedin-U	P48645	(NMU_HUMAN)
CDC45	Cell division control protein 45 homolog	O75419	(CDC45_HUMAN)
MCM2	DNA replication licensing factor MCM2	P49736	(MCM2_HUMAN)
SPP1	Osteopontin	P10451	(OSTP_HUMAN)
ASPM	Abnormal spindle-like microcephaly-associated protein	Q8IZT6	(ASPM_HUMAN)
RBM28	RNA-binding protein 28	Q9NW13	(RBM28_HUMAN)
HEATR1	HEAT repeat-containing protein 1	Q9H583	(HEAT1_HUMAN)
WDR75	WD repeat-containing protein 75	Q8IWA0	(WDR75_HUMAN)
CCR2	C-C chemokine receptor type 2	P41597	(CCR2_HUMAN)
GNG2	Guanine nucleotide-binding protein G(I)/G(S)/G(O) subunit gamma-2	P59768	(GBG2_HUMAN)
TMEM270	Transmembrane protein 270	Q6UE05	(TM270_HUMAN)
ACAA2	3-ketoacyl-CoA thiolase, mitochondrial	P42765	(THIM_HUMAN)
PGPEP1L	Pyroglutamyl-peptidase 1-like protein	A6NFU8	(PGPIL_HUMAN)
NOP58	Nucleolar protein 58	Q9Y2X3	(NOP58_HUMAN)
WDR3	WD repeat-containing protein 3	Q9UNX4	(WDR3_HUMAN)
RPP40	Ribonuclease P protein subunit p40	O75818	(RPP40_HUMAN)
GTPBP4	GTP-binding protein 4	Q9BZE4	(GTPB4_HUMAN)
WDR43	WD repeat-containing protein 43	Q15061	(WDR43_HUMAN)
POLR1C	DNA-directed RNA polymerases I and III subunit RPAC1	O15160	(RPAC1_HUMAN)
POLR1D	DNA-directed RNA polymerases I and III subunit RPAC2	P0DPB6	(RPAC2_HUMAN)
METTL3	N6-adenosine-methyltransferase catalytic subunit	Q86U44	(MTA70_HUMAN)

Table S2. Top Biological pathways among the 102 CRC markers.

Gene	Pathway counts*	GO - Biological Process**
**NDRG1**	**11**	GO:0007165 [signal transduction]
	**10**	GO:0008285 [negative regulation of cell proliferation]
	**7**	GO:0071456 [cellular response to hypoxia]
	**1**	GO:0010038 [response to metal ion]
	**1**	GO:0032287 [peripheral nervous system myelin maintenance]
**PRKACV**	-	-
**CAB39**	**11**	GO:0007165 [signal transduction]
	**5**	GO:0018105 [peptidyl-serine phosphorylation]
	**5**	GO:0035556 [intracellular signal transduction]
	**2**	GO:0032147 [activation of protein kinase activity]
	**2**	GO:0014823 [response to activity]
**CFLAR**	**13**	GO:0043066 [negative regulation of apoptotic process]
	**8**	GO:0006915 [apoptotic process]
	**7**	GO:0071456 [cellular response to hypoxia]
	**4**	GO:0006508 [proteolysis]
	**4**	GO:2001237 [negative regulation of extrinsic apoptotic signaling pathway]
**CTSC**	**8**	GO:0006915 [apoptotic process]
	**6**	GO:0007568 [aging]
	**5**	GO:0006955 [immune response]
	**4**	GO:0006508 [proteolysis]
	**2**	GO:0010033 [response to organic substance]
**THBS1**	**13**	GO:0043066 [negative regulation of apoptotic process]
	**11**	GO:0008284 [positive regulation of cell proliferation]
	**10**	GO:0006954 [inflammatory response]
	**10**	GO:0008285 [negative regulation of cell proliferation]
	**8**	GO:0009410 [response to xenobiotic stimulus]
**TRAPPC3**	**1**	GO:0006888 [ER to Golgi vesicle-mediated transport]
	**1**	GO:0006891 [intra-Golgi vesicle-mediated transport]
**EIF4E3**	**1**	GO:0006413 [translational initiation]
	**1**	GO:0006417 [regulation of translation]
**PTK6**	**8**	GO:0006468 [protein phosphorylation]
	**4**	GO:0046777 [protein autophosphorylation]
	**3**	GO:0042531 [positive regulation of tyrosine phosphorylation of STAT protein]
	**3**	GO:0030154 [cell differentiation]
	**3**	GO:0071300 [cellular response to retinoic acid]
**PHLPP2**	**11**	GO:0007165 [signal transduction]
	**2**	GO:0021766 [hippocampus development]
	**1**	GO:0006470 [protein dephosphorylation]
**CBX7**	**8**	GO:0009410 [response to xenobiotic stimulus]
	**8**	GO:0000122 [negative regulation of transcription from RNA polymerase II promoter]
	**1**	GO:0032968 [positive regulation of transcription elongation from RNA polymerase II promoter]
	**1**	GO:0006325 [chromatin organization]
	**1**	GO:0003006 [developmental process involved in reproduction]
**LPAR1**	**10**	GO:0007186 [G-protein coupled receptor signaling pathway]
	**4**	GO:0007189 [adenylate cyclase-activating G-protein coupled receptor signaling pathway]
	**4**	GO:0060326 [cell chemotaxis]
	**3**	GO:0043123 [positive regulation of I-kappaB kinase/NF-kappaB signaling]
	**2**	GO:0043410 [positive regulation of MAPK cascade]

**RCL1**	**2**	GO:0000447 [endonucleolytic cleavage in ITS1 to separate SSU-rRNA from 5.8S rRNA and LSU-rRNA from tricistronic rRNA transcript (SSU-rRNA, 5.8S rRNA, LSU-rRNA)]
	**1**	GO:0000479 [endonucleolytic cleavage of tricistronic rRNA transcript (SSU-rRNA, 5.8S rRNA, LSU-rRNA)]
	**1**	GO:0008150 [biological_process]
	**1**	GO:0000480 [endonucleolytic cleavage in 5'-ETS of tricistronic rRNA transcript (SSU-rRNA, 5.8S rRNA, LSU-rRNA)]
**ZG16**	**1**	GO:0050830 [defense response to Gram-positive bacterium]
	**1**	GO:0052373 [negative regulation by organism of entry into other organism involved in symbiotic interaction]
	**1**	GO:0015031 [protein transport]
**ECHS1**	**2**	GO:0006635 [fatty acid beta-oxidation]
	**1**	GO:0009083 [branched-chain amino acid catabolic process]
**TOP2A**	**11**	GO:0045944 [positive regulation of transcription from RNA polymerase II promoter]
	**6**	GO:0006974 [cellular response to DNA damage stimulus]
	**4**	GO:0043065 [positive regulation of apoptotic process]
	**4**	GO:0007059 [chromosome segregation]
	**2**	GO:0007143 [female meiotic division]
**CXCL12**	**11**	GO:0007165 [signal transduction]
	**10**	GO:0007186 [G-protein coupled receptor signaling pathway]
	**8**	GO:0070098 [chemokine-mediated signaling pathway]
	**5**	GO:0006955 [immune response]
	**5**	GO:0030335 [positive regulation of cell migration]
**CKS2**	**12**	GO:0051301 [cell division]
	**9**	GO:0006357 [regulation of transcription from RNA polymerase II promoter]
	**7**	GO:0008283 [cell proliferation]
	**4**	GO:0007346 [regulation of mitotic cell cycle]
	**3**	GO:0044772 [mitotic cell cycle phase transition]
**TTK**	**11**	GO:0008284 [positive regulation of cell proliferation]
	**6**	GO:0007052 [mitotic spindle organization]
	**5**	GO:0018105 [peptidyl-serine phosphorylation]
	**4**	GO:0007094 [mitotic spindle assembly checkpoint]
	**4**	GO:0007059 [chromosome segregation]
**NHP2**	**2**	GO:0000469 [cleavage involved in rRNA processing]
	**1**	GO:0000470 [maturation of LSU-rRNA]
	**1**	GO:0000454 [snoRNA guided rRNA pseudouridine synthesis]
	**1**	GO:0031120 [snRNA pseudouridine synthesis]
	**1**	GO:1904874 [positive regulation of telomerase RNA localization to Cajal body]
**RAN**	**12**	GO:0051301 [cell division]
	**6**	GO:0007052 [mitotic spindle organization]
	**5**	GO:0000278 [mitotic cell cycle]
	**4**	GO:0000070 [mitotic sister chromatid segregation]
	**3**	GO:0042307 [positive regulation of protein import into nucleus]
**FOXO1**	**13**	GO:0043066 [negative regulation of apoptotic process]
	**11**	GO:0045944 [positive regulation of transcription from RNA polymerase II promoter]
	**9**	GO:0006357 [regulation of transcription from RNA polymerase II promoter]
	**9**	GO:0045893 [positive regulation of transcription, DNA-templated]
	**8**	GO:0000122 [negative regulation of transcription from RNA polymerase II promoter]
**CDC6**	**12**	GO:0051301 [cell division]
	**10**	GO:0008285 [negative regulation of cell proliferation]
	**6**	GO:0048146 [positive regulation of fibroblast proliferation]
	**5**	GO:0000278 [mitotic cell cycle]
	**4**	GO:0000079 [regulation of cyclin-dependent protein serine/threonine kinase activity]

**YAP1**	**11**	GO:0045944 [positive regulation of transcription from RNA polymerase II promoter]
	**9**	GO:0010628 [positive regulation of gene expression]
	**9**	GO:0010629 [negative regulation of gene expression]
	**9**	GO:0045893 [positive regulation of transcription, DNA-templated]
	**8**	GO:0000122 [negative regulation of transcription from RNA polymerase II promoter]
**CDK1**	**13**	GO:0043066 [negative regulation of apoptotic process]
	**12**	GO:0051301 [cell division]
	**9**	GO:0010629 [negative regulation of gene expression]
	**9**	GO:0010628 [positive regulation of gene expression]
	**8**	GO:0009410 [response to xenobiotic stimulus]
**VEGFA**	**13**	GO:0043066 [negative regulation of apoptotic process]
	**11**	GO:0008284 [positive regulation of cell proliferation]
	**11**	GO:0045944 [positive regulation of transcription from RNA polymerase II promoter]
	**9**	GO:0010629 [negative regulation of gene expression]
	**9**	GO:0010628 [positive regulation of gene expression]
**CCNB1**	**12**	GO:0051301 [cell division]
	**8**	GO:0006468 [protein phosphorylation]
	**6**	GO:0048146 [positive regulation of fibroblast proliferation]
	**6**	GO:0007052 [mitotic spindle organization]
	**5**	GO:0001701 [in utero embryonic development]
**DLGAP5**	**6**	GO:0007052 [mitotic spindle organization]
	**4**	GO:0007346 [regulation of mitotic cell cycle]
	**4**	GO:0007059 [chromosome segregation]
	**2**	GO:0051642 [centrosome localization]
	**1**	GO:0023052 [signaling]
**CXCL1**	**11**	GO:0007165 [signal transduction]
	**10**	GO:0007186 [G-protein coupled receptor signaling pathway]
	**10**	GO:0006954 [inflammatory response]
	**10**	GO:0008285 [negative regulation of cell proliferation]
	**8**	GO:0070098 [chemokine-mediated signaling pathway]
**ADCY9**	**11**	GO:0007165 [signal transduction]
	**5**	GO:0001701 [in utero embryonic development]
	**5**	GO:0035556 [intracellular signal transduction]
	**4**	GO:0007189 [adenylate cyclase-activating G-protein coupled receptor signaling pathway]
	**1**	GO:0071880 [adenylate cyclase-activating adrenergic receptor signaling pathway]
**NEK2**	**12**	GO:0051301 [cell division]
	**8**	GO:0006468 [protein phosphorylation]
	**5**	GO:0000278 [mitotic cell cycle]
	**4**	GO:0007059 [chromosome segregation]
	**4**	GO:0046777 [protein autophosphorylation]
**NOB1**	**5**	GO:0006364 [rRNA processing]
	**2**	GO:0000469 [cleavage involved in rRNA processing]
	**2**	GO:0030490 [maturation of SSU-rRNA]
	**1**	GO:0090502 [RNA phosphodiester bond hydrolysis, endonucleolytic]
	**1**	GO:0007601 [visual perception]
**CCL21**	**10**	GO:0006954 [inflammatory response]
	**10**	GO:0007186 [G-protein coupled receptor signaling pathway]
	**8**	GO:0070098 [chemokine-mediated signaling pathway]
	**6**	GO:0007267 [cell-cell signaling]
	**6**	GO:0030593 [neutrophil chemotaxis]
**PTTG1**	**12**	GO:0051301 [cell division]
	**4**	GO:0006281 [DNA repair]
	**4**	GO:0007283 [spermatogenesis]
	**4**	GO:0010951 [negative regulation of endopeptidase activity]
	**2**	GO:0051276 [chromosome organization]

**SERPINE1**	**7**	GO:0071222 [cellular response to lipopolysaccharide]
	**6**	GO:0001525 [angiogenesis]
	**4**	GO:0045766 [positive regulation of angiogenesis]
	**4**	GO:0010951 [negative regulation of endopeptidase activity]
	**3**	GO:0090026 [positive regulation of monocyte chemotaxis]
**PLK1**	**13**	GO:0043066 [negative regulation of apoptotic process]
	**8**	GO:0000122 [negative regulation of transcription from RNA polymerase II promoter]
	**8**	GO:0006468 [protein phosphorylation]
	**6**	GO:0007052 [mitotic spindle organization]
	**5**	GO:0000086 [G2/M transition of mitotic cell cycle]
**MYC**	**13**	GO:0043066 [negative regulation of apoptotic process]
	**11**	GO:0008284 [positive regulation of cell proliferation]
	**11**	GO:0045944 [positive regulation of transcription from RNA polymerase II promoter]
	**9**	GO:0010628 [positive regulation of gene expression]
	**9**	GO:0045893 [positive regulation of transcription, DNA-templated]
**RRM2**	**5**	GO:0006260 [DNA replication]
	**2**	GO:0001824 [blastocyst development]
	**1**	GO:0009263 [deoxyribonucleotide biosynthetic process]
	**1**	GO:0051290 [protein heterotetramerization]
	**1**	GO:0009185 [ribonucleoside diphosphate metabolic process]
**IGF1**	**13**	GO:0043066 [negative regulation of apoptotic process]
	**11**	GO:0008284 [positive regulation of cell proliferation]
	**11**	GO:0007165 [signal transduction]
	**11**	GO:0045944 [positive regulation of transcription from RNA polymerase II promoter]
	**9**	GO:0010629 [negative regulation of gene expression]
**CHEK1**	**8**	GO:0006468 [protein phosphorylation]
	**8**	GO:0006915 [apoptotic process]
	**6**	GO:0006974 [cellular response to DNA damage stimulus]
	**5**	GO:0000086 [G2/M transition of mitotic cell cycle]
	**5**	GO:0006260 [DNA replication]
**GINS1**		-
**NLRP7**	**7**	GO:0071222 [cellular response to lipopolysaccharide]
	**4**	GO:0010951 [negative regulation of endopeptidase activity]
	**4**	GO:0071347 [cellular response to interleukin-1]
	**2**	GO:0032691 [negative regulation of interleukin-1 beta production]
	**1**	GO:0010955 [negative regulation of protein processing]
**EREG**	**11**	GO:0008284 [positive regulation of cell proliferation]
	**10**	GO:0008285 [negative regulation of cell proliferation]
	**6**	GO:0048146 [positive regulation of fibroblast proliferation]
	**6**	GO:0001525 [angiogenesis]
	**6**	GO:0007267 [cell-cell signaling]
**UTP14A**	**5**	GO:0006364 [rRNA processing]
**ANTXR1**	**2**	GO:0001568 [blood vessel development]
	**1**	GO:0034446 [substrate adhesion-dependent cell spreading]
	**1**	GO:1901998 [toxin transport]
	**1**	GO:0031532 [actin cytoskeleton reorganization]
	**1**	GO:1901202 [negative regulation of extracellular matrix assembly]
**ASF1A**	**4**	GO:0006281 [DNA repair]
	**3**	GO:0001649 [osteoblast differentiation]
	**2**	GO:0006334 [nucleosome assembly]
	**1**	GO:0006335 [DNA replication-dependent nucleosome assembly]
	**1**	GO:0006336 [DNA replication-independent nucleosome assembly]

**TIMP1**	**13**	GO:0043066 [negative regulation of apoptotic process]
	**11**	GO:0007165 [signal transduction]
	**11**	GO:0008284 [positive regulation of cell proliferation]
	**6**	GO:0007568 [aging]
	**4**	GO:0010951 [negative regulation of endopeptidase activity]
**CEP55**	**2**	GO:0045184 [establishment of protein localization]
	**2**	GO:0007080 [mitotic metaphase plate congression]
	**2**	GO:0006997 [nucleus organization]
	**2**	GO:0000281 [mitotic cytokinesis]
	**1**	GO:0014066 [regulation of phosphatidylinositol 3-kinase signaling]
**SFTA2**		-
**AMH**	**9**	GO:0010628 [positive regulation of gene expression]
	**8**	GO:0009410 [response to xenobiotic stimulus]
	**6**	GO:0007568 [aging]
	**6**	GO:0007267 [cell-cell signaling]
	**4**	GO:0001541 [ovarian follicle development]
**MYH2**	**1**	GO:0006936 [muscle contraction]
	**1**	GO:0030049 [muscle filament sliding]
**POU4F1**	**13**	GO:0043066 [negative regulation of apoptotic process]
	**11**	GO:0045944 [positive regulation of transcription from RNA polymerase II promoter]
	**9**	GO:0006357 [regulation of transcription from RNA polymerase II promoter]
	**9**	GO:0010628 [positive regulation of gene expression]
	**9**	GO:0010629 [negative regulation of gene expression]
**SIX4**	**13**	GO:0043066 [negative regulation of apoptotic process]
	**11**	GO:0045944 [positive regulation of transcription from RNA polymerase II promoter]
	**9**	GO:0006357 [regulation of transcription from RNA polymerase II promoter]
	**9**	GO:0045893 [positive regulation of transcription, DNA-templated]
	**3**	GO:0008584 [male gonad development]
**INHBA**	**11**	GO:0045944 [positive regulation of transcription from RNA polymerase II promoter]
	**10**	GO:0008285 [negative regulation of cell proliferation]
	**9**	GO:0010628 [positive regulation of gene expression]
	**9**	GO:0045893 [positive regulation of transcription, DNA-templated]
	**9**	GO:0006357 [regulation of transcription from RNA polymerase II promoter]
**ESM1**	**11**	GO:0008284 [positive regulation of cell proliferation]
	**6**	GO:0001525 [angiogenesis]
	**3**	GO:0002040 [sprouting angiogenesis]
	**1**	GO:1902204 [positive regulation of hepatocyte growth factor receptor signaling pathway]
**SLC10A1**	**4**	GO:0045471 [response to ethanol]
	**3**	GO:0046718 [viral entry into host cell]
	**3**	GO:0010468 [regulation of gene expression]
	**2**	GO:0071466 [cellular response to xenobiotic stimulus]
	**2**	GO:0055085 [transmembrane transport]
**MAPT**	**9**	GO:0010629 [negative regulation of gene expression]
	**6**	GO:0007267 [cell-cell signaling]
	**3**	GO:0034605 [cellular response to heat]
	**2**	GO:0031175 [neuron projection development]
	**2**	GO:0007613 [memory]
**DDIT4**	**8**	GO:0006915 [apoptotic process]
	**4**	GO:0001666 [response to hypoxia]
	**3**	GO:0007420 [brain development]
	**3**	GO:0001764 [neuron migration]
	**2**	GO:0071549 [cellular response to dexamethasone stimulus]
**TPTE**		-

**PAICS**	**1**	GO:0006189 ['de novo' IMP biosynthetic process]
	**1**	GO:0006177 [GMP biosynthetic process]
	**1**	GO:0044208 ['de novo' AMP biosynthetic process]
	**1**	GO:0097294 ['de novo' XMP biosynthetic process]
	**1**	GO:0009113 [purine nucleobase biosynthetic process]
**CCNA2**	**12**	GO:0051301 [cell division]
	**9**	GO:0045893 [positive regulation of transcription, DNA-templated]
	**7**	GO:0071456 [cellular response to hypoxia]
	**6**	GO:0048146 [positive regulation of fibroblast proliferation]
	**5**	GO:0000086 [G2/M transition of mitotic cell cycle]
**PAX5**	**11**	GO:0045944 [positive regulation of transcription from RNA polymerase II promoter]
	**9**	GO:0006357 [regulation of transcription from RNA polymerase II promoter]
	**8**	GO:0000122 [negative regulation of transcription from RNA polymerase II promoter]
	**6**	GO:0007568 [aging]
	**4**	GO:0007283 [spermatogenesis]
**CXCL3**	**10**	GO:0006954 [inflammatory response]
	**8**	GO:0070098 [chemokine-mediated signaling pathway]
	**7**	GO:0071222 [cellular response to lipopolysaccharide]
	**6**	GO:0030593 [neutrophil chemotaxis]
	**5**	GO:0061844 [antimicrobial humoral immune response mediated by antimicrobial peptide]
**CXCL11**	**11**	GO:0007165 [signal transduction]
	**10**	GO:0006954 [inflammatory response]
	**8**	GO:0070098 [chemokine-mediated signaling pathway]
	**7**	GO:0071222 [cellular response to lipopolysaccharide]
	**6**	GO:0007267 [cell-cell signaling]
**PPBP**	**10**	GO:0006954 [inflammatory response]
	**8**	GO:0070098 [chemokine-mediated signaling pathway]
	**7**	GO:0071222 [cellular response to lipopolysaccharide]
	**6**	GO:0030593 [neutrophil chemotaxis]
	**5**	GO:0061844 [antimicrobial humoral immune response mediated by antimicrobial peptide]
	**1**	GO:0042742 [defense response to bacterium]
**CDC20**	**12**	GO:0051301 [cell division]
	**11**	GO:0008284 [positive regulation of cell proliferation]
	**4**	GO:0007399 [nervous system development]
	**3**	GO:0030154 [cell differentiation]
	**2**	GO:0031915 [positive regulation of synaptic plasticity]
**MCM4**	**5**	GO:0006260 [DNA replication]
	**3**	GO:0006268 [DNA unwinding involved in DNA replication]
	**3**	GO:0000727 [double-strand break repair via break-induced replication]
	**2**	GO:1902975 [mitotic DNA replication initiation]
	**1**	GO:0006271 [DNA strand elongation involved in DNA replication]
**CLDN1**	**6**	GO:0007568 [aging]
	**5**	GO:0030335 [positive regulation of cell migration]
	**4**	GO:0007155 [cell adhesion]
	**4**	GO:0071356 [cellular response to tumor necrosis factor]
	**4**	GO:0045471 [response to ethanol]
**SHANK2**	**11**	GO:0008284 [positive regulation of cell proliferation]
	**8**	GO:0009410 [response to xenobiotic stimulus]
	**3**	GO:0007420 [brain development]
	**2**	GO:0008344 [adult locomotory behavior]
	**2**	GO:0030534 [adult behavior]

**PTH1R**	**11**	GO:0008284 [positive regulation of cell proliferation]
	**10**	GO:0008285 [negative regulation of cell proliferation]
	**10**	GO:0007186 [G-protein coupled receptor signaling pathway]
	**5**	GO:0001701 [in utero embryonic development]
	**4**	GO:0007189 [adenylate cyclase-activating G-protein coupled receptor signaling pathway]
**C2**	**4**	GO:0006508 [proteolysis]
	**3**	GO:0032496 [response to lipopolysaccharide]
	**3**	GO:0045087 [innate immune response]
	**2**	GO:0097066 [response to thyroid hormone]
	**1**	GO:0006956 [complement activation]
**TEAD4**	**11**	GO:0045944 [positive regulation of transcription from RNA polymerase II promoter]
	**9**	GO:0006357 [regulation of transcription from RNA polymerase II promoter]
	**4**	GO:0007566 [embryo implantation]
	**3**	GO:0001501 [skeletal system development]
	**2**	GO:0007517 [muscle organ development]
**CXCL8**	**11**	GO:0007165 [signal transduction]
	**10**	GO:0007186 [G-protein coupled receptor signaling pathway]
	**10**	GO:0008285 [negative regulation of cell proliferation]
	**10**	GO:0006954 [inflammatory response]
	**9**	GO:0010629 [negative regulation of gene expression]
**AQP8**	**2**	GO:0055085 [transmembrane transport]
	**1**	GO:0071320 [cellular response to cAMP]
	**1**	GO:0006833 [water transport]
**PBX4**	**9**	GO:0045893 [positive regulation of transcription, DNA-templated]
	**9**	GO:0006357 [regulation of transcription from RNA polymerase II promoter]
	**3**	GO:0007420 [brain development]
	**3**	GO:0009887 [animal organ morphogenesis]
	**2**	GO:0048568 [embryonic organ development]
**POLR1B**	**4**	GO:0007566 [embryo implantation]
	**1**	GO:0014029 [neural crest formation]
	**1**	GO:0017126 [nucleologenesis]
	**1**	GO:0009303 [rRNA transcription]
**BUB1**	**12**	GO:0051301 [cell division]
	**8**	GO:0006915 [apoptotic process]
	**8**	GO:0006468 [protein phosphorylation]
	**7**	GO:0008283 [cell proliferation]
	**4**	GO:0007094 [mitotic spindle assembly checkpoint]
**AURKA**	**13**	GO:0043066 [negative regulation of apoptotic process]
	**12**	GO:0051301 [cell division]
	**9**	GO:0010629 [negative regulation of gene expression]
	**8**	GO:0006468 [protein phosphorylation]
	**6**	GO:0007052 [mitotic spindle organization]
**GNG7**	**10**	GO:0007186 [G-protein coupled receptor signaling pathway]
	**1**	GO:0008277 [regulation of G-protein coupled receptor protein signaling pathway]
**MAD2L1**	**13**	GO:0043066 [negative regulation of apoptotic process]
	**12**	GO:0051301 [cell division]
	**4**	GO:0007094 [mitotic spindle assembly checkpoint]
	**4**	GO:0000070 [mitotic sister chromatid segregation]
	**2**	GO:0051444 [negative regulation of ubiquitin-protein transferase activity]
**PTGS2**	**10**	GO:0006954 [inflammatory response]
	**10**	GO:0008285 [negative regulation of cell proliferation]
	**8**	GO:0009410 [response to xenobiotic stimulus]
	**7**	GO:0071456 [cellular response to hypoxia]
	**6**	GO:0001525 [angiogenesis]

**NMU**	**10**	GO:0007186 [G-protein coupled receptor signaling pathway]
	**2**	GO:0097009 [energy homeostasis]
	**2**	GO:0045987 [positive regulation of smooth muscle contraction]
	**2**	GO:0001659 [temperature homeostasis]
	**1**	GO:0060259 [regulation of feeding behavior]
**CDC45**	**3**	GO:0006270 [DNA replication initiation]
	**3**	GO:0000727 [double-strand break repair via break-induced replication]
	**3**	GO:0006268 [DNA unwinding involved in DNA replication]
	**2**	GO:0000076 [DNA replication checkpoint]
	**1**	GO:1902977 [mitotic DNA replication preinitiation complex assembly]
**MCM2**	**8**	GO:0006915 [apoptotic process]
	**5**	GO:0006260 [DNA replication]
	**3**	GO:0006270 [DNA replication initiation]
	**3**	GO:0000727 [double-strand break repair via break-induced replication]
	**3**	GO:0006268 [DNA unwinding involved in DNA replication]
**SPP1**	**11**	GO:0007165 [signal transduction]
	**10**	GO:0006954 [inflammatory response]
	**9**	GO:0045893 [positive regulation of transcription, DNA-templated]
	**4**	GO:0007566 [embryo implantation]
	**4**	GO:0007155 [cell adhesion]
**ASPM**	**4**	GO:0007283 [spermatogenesis]
	**3**	GO:0008584 [male gonad development]
	**3**	GO:0001764 [neuron migration]
	**3**	GO:0007051 [spindle organization]
	**2**	GO:0021987 [cerebral cortex development]
**RBM28**	**2**	GO:0006397 [mRNA processing]
	**1**	GO:0008380 [RNA splicing]
**HEATR1**	**3**	GO:2000234 [positive regulation of rRNA processing]
	**3**	GO:0045943 [positive regulation of transcription from RNA polymerase I promoter]
	**2**	GO:0000462 [maturation of SSU-rRNA from tricistronic rRNA transcript (SSU-rRNA, 5.8S rRNA, LSU-rRNA)]
**WDR75**	**5**	GO:0006364 [rRNA processing]
	**3**	GO:0045943 [positive regulation of transcription from RNA polymerase I promoter]
	**3**	GO:2000234 [positive regulation of rRNA processing]
**CCR2**	**10**	GO:0007186 [G-protein coupled receptor signaling pathway]
	**10**	GO:0006954 [inflammatory response]
	**8**	GO:0070098 [chemokine-mediated signaling pathway]
	**5**	GO:0006955 [immune response]
	**5**	GO:0006935 [chemotaxis]
**GNG2**	**10**	GO:0007186 [G-protein coupled receptor signaling pathway]
	**7**	GO:0008283 [cell proliferation]
	**1**	GO:0071870 [cellular response to catecholamine stimulus]
	**1**	GO:0071380 [cellular response to prostaglandin E stimulus]
	**1**	GO:0007191 [adenylate cyclase-activating dopamine receptor signaling pathway]
**TMEM270**	-	-
**ACAA2**	**7**	GO:0071456 [cellular response to hypoxia]
	**2**	GO:0006635 [fatty acid beta-oxidation]
	**1**	GO:0006695 [cholesterol biosynthetic process]
	**1**	GO:1901029 [negative regulation of mitochondrial outer membrane permeabilization involved in apoptotic signaling pathway]
	**1**	GO:1902109 [negative regulation of mitochondrial membrane permeability involved in apoptotic process]
**PGPEP1L**	**4**	GO:0006508 [proteolysis]
**NOP58**	**5**	GO:0006364 [rRNA processing]
	**1**	GO:0048254 [snoRNA localization]

**WDR3**	**2**	GO:0030490 [maturation of SSU-rRNA]
**RPP40**	**2**	GO:0000447 [endonucleolytic cleavage in ITS1 to separate SSU-rRNA from 5.8S rRNA and LSU-rRNA from tricistronic rRNA transcript (SSU-rRNA, 5.8S rRNA, LSU-rRNA)]
	**1**	GO:1905267 [endonucleolytic cleavage involved in tRNA processing]
	**1**	GO:0001682 [tRNA 5'-leader removal]
**GTPBP4**	**10**	GO:0008285 [negative regulation of cell proliferation]
	**4**	GO:0000079 [regulation of cyclin-dependent protein serine/threonine kinase activity]
	**3**	GO:0001649 [osteoblast differentiation]
	**2**	GO:0050821 [protein stabilization]
	**2**	GO:0030336 [negative regulation of cell migration]
**WDR43**	**5**	GO:0006364 [rRNA processing]
	**3**	GO:0045943 [positive regulation of transcription from RNA polymerase I promoter]
	**3**	GO:2000234 [positive regulation of rRNA processing]
	**2**	GO:0000462 [maturation of SSU-rRNA from tricistronic rRNA transcript (SSU-rRNA, 5.8S rRNA, LSU-rRNA)]
	**1**	GO:0034243 [regulation of transcription elongation from RNA polymerase II promoter]
**POLR1C**	**1**	GO:0006360 [transcription from RNA polymerase I promoter]
	**1**	GO:0006383 [transcription from RNA polymerase III promoter]

* The "pathway counts" is the number of times each stated pathway was counted among all the biological pathways to which any of the individual genes listed in Tables 1 and 2 belong.

** Up to 5 pathways shown.

Table S3. Downregulated genes in CRC.

Gene	Protein	UniProt ID	Protein Name
NDRG1	N-myc downstream-regulated gene 1 protein	E5RK17	(E5RK17_HUMAN)
PRKACV	cAMP-dependent protein kinase catalytic subunit beta	P22694	(KAPCB_HUMAN)
CAB39	Calcium-binding protein 39	Q9Y376	(CAB39_HUMAN)
CFLAR	CASP8 and FADD-like apoptosis regulator	O15519	(CFLAR_HUMAN)
CTSC	Cathepsin C	H0YCY8	(H0YCY8_HUMAN)
THBS1	Thrombospondin-1	P07996	(TSP1_HUMAN)
TRAPPC3	Trafficking protein particle complex subunit 3	O43617	(TPPC3_HUMAN)
EIF4E3	Eukaryotic translation initiation factor 4E type 3	Q8N5X7	(IF4E3_HUMAN)
PTK6	Protein-tyrosine kinase 6	Q13882	(PTK6_HUMAN)
PHLPP2	PH domain leucine-rich repeat-containing protein phosphatase 2	Q6ZVD8	(PHLP2_HUMAN)
CBX7	Chromobox protein homolog 7	O95931	(CBX7_HUMAN)
LPAR1	Lysophosphatidic acid receptor 1	Q92633	(LPAR1_HUMAN)
RCL1	RNA 3'-terminal phosphate cyclase-like protein	Q9Y2P8	(RCL1_HUMAN)
ZG16	Zymogen granule membrane protein 16	O60844	(ZG16_HUMAN)
